# A cluster randomised controlled trial of the efficacy of a brief walking intervention delivered in primary care: Study protocol

**DOI:** 10.1186/1471-2296-12-56

**Published:** 2011-06-23

**Authors:** David P French, Stefanie L Williams, Susan Michie, Claire Taylor, Ala Szczepura, Nigel Stallard, Jeremy Dale

**Affiliations:** 1 Applied Research Centre in Health and Lifestyle Interventions, Faculty of Health and Life Sciences, Coventry University, Priory Street, Coventry CV1 5FB, UK; 2 Department of Clinical, Educational and Health Psychology, University College London, 1-19 Torrington Place, London WC1E 7HB, UK; 3 Clinical Sciences Research Institute, Warwick Medical School, Social Studies Building, Main Campus, Coventry, CV4 7AL, UK; 4 Health Sciences Research Institute, Warwick Medical School, Gibbet Hill Campus, University of Warwick, Coventry, CV4 7AL, UK

## Abstract

**Background:**

The aim of the present research is to conduct a fully powered explanatory trial to evaluate the efficacy of a brief self-regulation intervention to increase walking. The intervention will be delivered in primary care by practice nurses (PNs) and Healthcare Assistants (HCAs) to patients for whom increasing physical activity is a particular priority. The intervention has previously demonstrated efficacy with a volunteer population, and subsequently went through an iterative process of refinement in primary care, to maximise acceptability to both providers and recipients.

**Methods/ Design:**

This two arm cluster randomised controlled trial set in UK general practices will compare two strategies for increasing walking, assessed by pedometer, over six months. Patients attending practices randomised to the self-regulation intervention arm will receive an intervention consisting of behaviour change techniques designed to increase walking self-efficacy (confidence in ability to perform the behaviour), and to help people translate their "good" intentions into behaviour change by making plans. Patients attending practices randomised to the information provision arm will receive written materials promoting walking, and a short unstructured discussion about increasing their walking.

The trial will recruit 20 PN/HCAs (10 per arm), who will be trained by the research team to deliver the self-regulation intervention or information provision control intervention, to 400 patients registered at their practices (20 patients per PN/HCA). This will provide 85% power to detect a mean difference of five minutes/day walking between the self-regulation intervention group and the information provision control group. Secondary outcomes include health services costs, and intervention effects in sub-groups defined by age, ethnicity, gender, socio-economic status, and clinical condition. A mediation analysis will investigate the extent to which changes in constructs specified by the Theory of Planned Behaviour lead to changes in objectively assessed walking behaviour.

**Discussion:**

This trial addresses the current lack of evidence for interventions that are effective at increasing walking and that can be offered to patients in primary care. The intervention being evaluated has demonstrated efficacy, and has been through an extensive process of adaptation to ensure acceptability to both provider and recipient, thus optimising fidelity of intervention delivery and treatment receipt. It therefore provides a strong test of the hypothesis that a self-regulation intervention can help primary care patients increase their walking.

**Trial registration:**

Current Controlled Trials ISRCTN95932902

## Background

The Department of Health for England currently recommends that individuals should engage in at least 30 minutes of "moderate or vigorous" exercise, for at least five days each week, to gain health protective benefits [1]. Over 60% of men and 70% of women in England and Wales do not report meeting these requirements, and the proportion who actually meet them is likely to be considerably lower [2]. The Chief Medical Officer for England has recognised that many people have difficulty in translating a physical activity recommendation into daily life [3].

Brisk walking (i.e. walking of sufficient intensity to result in the walker getting slightly out of breath) is a form of moderate physical activity which is especially promising for health promotion because of its acceptability and accessibility, particularly among populations who are the most physically inactive [4]. It does not have to be scheduled, nor does it require any special clothes or equipment, so it is relatively low cost. Further, walking is low impact so minimises the risk of injuries and does not cause as much anxiety regarding adverse effects in sedentary populations as more vigorous forms of physical activity. There is meta-analytic evidence that increases in walking in previously sedentary adults increase fitness, and decrease body weight, BMI, percentage body fat and resting diastolic blood pressure [5]. That is, not only is walking acceptable to sedentary people, but it affords them considerable health benefits.

A recent systematic review and meta-analysis of 19 randomised and 29 non-randomised controlled studies provided evidence of the potential efficacy of walking interventions [6]. It concluded that "the most successful interventions could increase walking among targeted participants by up to 30-60 minutes a week on average, at least in the short term" [6]. The usefulness of this review was limited by including a mix of different types of interventions targeted at different populations, using different modes of delivery and different assessments of change in walking, which makes it difficult to identify the most effective intervention strategies. In addition the extent to which these interventions were sustainable, generalisable, or cost-effective is unclear, making interpretation to inform policy difficult.

A 2006 review from the National Institute of Health and Clinical Excellence (NICE) concluded that, although there is considerable evidence for the benefits that accrue from walking, there is a shortage of effective interventions that can be offered to patients in primary care [7]. This conclusion was unchanged when the review was updated in 2011. The advantages of a primary care setting are that this is where most of the population has regular contact with the healthcare system, and often in circumstances where they are receptive to advice to alter their behaviour. There has also been increased delivery of lifestyle advice by practice nurses (PNs) and healthcare assistants (HCAs), based in, or working closely with primary care. If an intervention to increase walking in primary care is effective and cost-effective, then primary care offers the possibilities of scaling this up to allow a large proportion of the population to receive the benefits of being more physically active.

The present study protocol describes a cluster randomised controlled trial of an intervention to increase walking in primary care which was designed to address the lack of effective interventions that can be offered to patients identified by NICE. In line with the Medical Research Council (MRC) framework for development and evaluation of complex interventions [8,9], the trial intervention and procedures build on previous formative research [10-14], based on explicit theory [15-17].

Extended developmental work was conducted using the Theory of Planned Behaviour (TPB), a social cognition model which can be used to predict and explain any behaviour in terms of a limited set of constructs [15]. A review of previous research using this theory identified perceived behavioural control (PBC) as the strongest predictor of intentions to walk more [13]. According to the TPB, PBC is underpinned by control beliefs: beliefs about which factors make it easy or difficult to perform the behaviour [15]. The most common control beliefs regarding walking in a general public sample [10] were identified as related to lack of time, indicating that the intervention should aim to address these beliefs. As PBC is a highly similar, if not identical, construct to self-efficacy [18], the literature on altering self-efficacy was consulted [16], which suggested that mastery experience was likely to be effective [16].

It is now well recognised that there is often a "gap" between intentions and behaviour [19]. Consequently, this intervention included not only a motivational component, to increase the intention to walk more, but also a volitional arm to enact those intentions [20]. This may be particularly important for walking, as the association between intentions and behaviour has been found to be low in past research [11]. This may be due to people reporting walking as being a means to achieve other goals, rather than a goal in itself, which is the case with many other forms of physical activity [12].

The formative research led to the development of a single session intervention of 15 minutes duration. It consists of techniques to increase self-efficacy (confidence in ability to perform the behaviour), and planning techniques to help people translate their "good intentions" into behaviour change [14]. This was done by, e.g. asking participants to recall previous walking successes [16] and by forming action plans about when, where, how and with whom they would increase their walking [17,20].

An evaluation of this intervention with 130 adult volunteers showed that it yielded large increases (Cohen's d = 0.90) in objectively assessed walking behaviour [14]. The mean increase of 87 minutes/week compares favourably with the mean increase of 30-60 minutes/week among the "most successful interventions" to promote walking identified by Ogilvie and colleagues [6]. These increases in objectively assessed behaviour were mediated by changes in walking self-efficacy, in line with the theoretical basis of the intervention, and were maintained for at least six weeks post-intervention. Further, similarly large effects on objectively assessed walking behaviour (d = 1.06) were obtained when the intervention was delivered to another volunteer sample by a person who was not involved in its development, indicating that the observed effects were due to the intervention itself, rather than the person delivering it [21]. The mechanism by which the intervention worked appeared again to be due to increasing self-efficacy, with little apparent effect of the intervention on self-reported planning [21].

The intervention was further developed following a subsequent meta-analysis that examined which intervention techniques [22] were associated with improvements in self-efficacy for increasing physical activity [23]. The technique of providing structured feedback was identified in the review as being associated with improvements in self-efficacy [23]. It was not included in the original intervention but has since been added to enhance the intervention's effectiveness. In addition, the technique of coping planning [17], which includes identification of barriers to physical activity, was removed, given that barrier identification was associated with poorer self-efficacy in the review. It was notable that those techniques which were associated with increases in self-efficacy were the same techniques which were associated with increases in physical activity (r = 0.69), providing further support for the idea that self-efficacy is a key determinant of such interventions [24].

The walking intervention has subsequently been extensively refined for delivery in primary care. A key aim of this refinement was to ensure provider fidelity of delivery to the intervention manual, and maximise recipient fidelity of receipt and enactment of the intervention techniques. If interventions are not delivered or received as intended (i.e. as per protocol) then it is difficult, if not impossible to be certain that the results can be attributed to the intervention itself [25]. This developmental work therefore tried to retain those key principles and elements of an intervention which had already demonstrated efficacy when delivered outside of a healthcare system to adult volunteers, and also optimise acceptability to both recipients and providers. Three phases of development work were conducted, before the current trial.

First, the intervention was revised, in an iterative fashion. A Research Nurse who was part of the research team delivered the intervention, based on an explicit manual, to patients in a single primary care practice within a local healthcare organisation, Coventry Primary Care Trust (PCT). Each intervention session was recorded, along with a brief interview with the intervention recipient conducted by a different research team member at the end of the intervention. On the basis of problems arising, the intervention was revised following discussion by the research team. The intervention was then further delivered, and revised, until it had been delivered to 10 patients, to optimise acceptability of the intervention to the patient recipients.

Secondly, two PNs and one HCA were trained, each delivering the intervention to four patients in their practices. The PN/HCAs were also interviewed prior to, and after, both the training and delivery to assess acceptability of the training and intervention materials. A sample of patients was also interviewed to establish comprehension and acceptability of the intervention. The training and intervention materials were then revised to optimise acceptability to PNs/ HCAs delivering the intervention.

Thirdly, an exploratory (pilot) trial was conducted, with 10 PNs/ HCAs recruited and trained, and eight delivering the intervention to up to 15 patients per practice. Two PNs were recruited and trained to deliver the control intervention to up to 15 patients per practice. In total, 66 intervention patients and 20 control patients participated. Data from three sources were collected during this pilot trial to assess treatment fidelity in relation to delivery and receipt of the intervention and to investigate reasons for variation in fidelity. Taped intervention sessions are being analysed to quantify the extent to which each intervention technique was delivered as specified by the intervention manual. Eight PN/HCAs were interviewed after they completed delivery of the intervention to investigate their experiences of delivering the intervention. Twelve patients were interviewed immediately after receiving the intervention, and three months later, to investigate patients' experiences of participating in the intervention, and specifically how the intervention was received.

The main outcome of this developmental work in primary care was that the intervention was refined, so as to be acceptable to both recipients and providers. We believe this work has provided a solid foundation of intervention development, based on an intervention with demonstrated efficacy in increasing objectively assessed walking and extensive adaptation to fit the primary care context, whilst retaining its theoretical underpinning. The next stage of this research is to examine the extent to which this developmental work has resulted in an intervention which can demonstrate efficacy in changing the walking behaviour of patients, when delivered in general practices by Practice Nurses or Health Care Assistants (PN/HCAs). In addition, it is unclear to what extent any intervention effects are sustainable and cost-effective, making interpretation to inform policy difficult.

The overall aims of the present research therefore, are to conduct a fully powered, definitive explanatory trial to:

(a) Assess the efficacy of the intervention in changing objectively assessed walking behaviour, for up to six months post-intervention.

(b) Identify key intervention mechanisms.

(c) Estimate the difference in the cost of the resources used by patients in each arm of the trial, and the cost-effectiveness of the intervention.

## Methods/Design

### Study Design

This explanatory trial is a two-arm cluster randomised controlled trial, with clustering by GP practice. It will assess differences in objectively assessed walking between patients who receive a brief self-regulation intervention, compared with patients who are provided with information about the benefits of walking. PNs/ HCAs randomised to the intervention group will deliver a brief self-regulation intervention to eligible patients, targeting self-efficacy and planning. PNs/ HCAs randomised to the "information provision" control group will provide information to eligible patients using materials which are currently widely available.

The study protocol was approved by Birmingham East, North and Solihull Research Ethics Committee (REC Ref: 09/H1206/116).

### Sample size

A total of 400 patients will be recruited from 20 PNs/HCAs (10 in each trial arm), each delivering the walking intervention sessions to 20 patients. Assuming a standard deviation of walking of 15 minutes/day, as obtained in a previous study with adult volunteers [14] and an intraclass correlation coefficient of 0.010, equal to the median value found in a review of clustering effects in practices in primary care [26], this will provide 85% power to detect a mean difference of five minutes/day walking between the intervention group, and the control group.

The difference in walking between groups that this explanatory trial is powered to detect is smaller than that observed in a previous study with adult volunteers [14] (12.4 mins/day over the first week, Cohen's d = 0.90) due to participants being primary care patients rather than volunteers, there being lower anticipated fidelity of treatment delivery, and a longer duration of outcome follow up.

### Setting/ participants

The study will take place in primary care practices in a geographically and socially diverse sub-region of central England (Coventry, Warwickshire, Worcestershire, and Herefordshire PCTs).

#### Practice Nurses/ Health Care Assistants

PNs/ HCAs in both the intervention and control arms will deliver sessions within the practices in which they are employed, to registered patients. All PNs/ HCAs who are randomised to the intervention group will be trained by the research team, and demonstrate competence to deliver the intervention, as assessed by the research team (details provided below) before delivering the intervention to patient participants. All PN/ HCAs randomised to the control group will be provided with one to one training on the control intervention and trial procedures from a member of the research team, usually involving one visit to their practice of approximately one hour. The PN/HCAs in the control arm will be offered the opportunity to receive training in the self-efficacy plus planning intervention after all participating patients from their practices have completed the 6 month follow-up measures.

#### Patients

To be eligible for inclusion in the trial, potential participants must be patients in a participating practice, as well as:

a) be aged between 16 and 65 years old,

b) have one of the following chronic conditions where physical activity has been shown to have a positive effect on health status [1]: hypertension, type 2 diabetes, pre-diabetes (impaired glucose tolerance, insulin resistance), low back pain, fibromyalgia, coronary heart disease, cardio-vascular disease, hypercholesterolemia, osteoporosis and osteoarthritis, or are overweight or obese,

c) not currently being investigated/treated by secondary care specialists for the condition (s) or are awaiting investigation/treatment by secondary care specialists for the condition (s),

d) are "sedentary" in terms of not meeting the 30 mins/day at least 5 times/ week guidelines [1],

e) are able to speak English (and therefore could potentially benefit from the PN/ HCA intervention session). This is because the intervention is to be delivered and interpreted verbally and there are not sufficient resources to employ interpreters. Where participants cannot adequately read English, the PN/HCA will read the patient information sheet, consent form and worksheet instructions.

f) do not have learning difficulties that would preclude active engagement with the intervention session,

g) do not have mental health problems i.e. anxiety, depression that would preclude active engagement with the intervention session, or where the patient is being investigated/treated by secondary care specialists for the problem,

h) wish to participate,

i) receive a letter of invitation to the study from their practice.

### Study intervention

#### Self-regulation intervention

Participants will receive the intervention in two face-to-face sessions, one week apart, with a telephone or face to face follow-up two weeks after the second session. The choice of face to face or telephone follow up will be decided by the PN/HCA in consultation with the patient, and will be dependent on the patient's and PN/HCA's own preference. Each session may be up to 30 minutes in duration, although it is anticipated that the follow-up session will be shorter. The intervention is fully detailed in the intervention manual, a copy of which is available on request from the first author.

Session one consists of motivational techniques to increase the patients' self-efficacy [16] about their ability to increase their walking, by requiring them to rehearse previous instances where they have successfully managed to complete long walks, or where they found such walks easy [27]. It also includes volitional techniques to facilitate realistic goal setting [28], and to translate intentions into practice (e.g. action plans) [17,20]. Session two consists of a review of progress since session one, involving positive feedback in relation to effort and achievement, and possible revision of goals set and action plans. It also includes the volitional technique of supportive/ facilitative planning, whereby the participant is encouraged to identify what factors support increased walking, and make plans to bring about those factors [14]. In this sense, it is similar to coping planning [17], but with a focus on how to increase and strengthen helpful factors, rather than how to overcome barriers. The follow-up session consists of an abbreviated version of some of the same techniques delivered in session two.

#### Training and fidelity assurance of self-regulation intervention

Each walking intervention PN/ HCA will be trained using the training manual and materials that have been developed, and refined in earlier phases of this research. All PN/HCAs will complete two training sessions, totalling seven hours. Training will involve formal presentations to introduce the PN/HCAs to the theory and research underpinning the intervention and the essential elements of the intervention: participative learning, practising delivery of intervention techniques, observation, feedback, discussion and homework. All PN/HCAs will become familiar with the intervention manual and materials. The training sessions will be conducted one week apart to allow PN/HCAs to absorb and reflect on what they have learnt and to re-read through the intervention materials.

After participating in the training sessions, PN/HCAs will be observed delivering both intervention sessions to either a colleague or acquaintance of the PN/HCA, or a researcher/ administration officer from Coventry University. Their competences will be assessed using 20-point checklists of intervention behaviours, rated by a member of the study team. To be certified competent in delivering the intervention, PN/HCAs need to achieve a minimum score of 15 out of a possible score of 20 for both sessions. PNs/HCAs will only deliver the intervention as part of the main trial after achieving this level of competence. Where the minimum level of competence is not achieved, the PN/HCA will be given feedback on potential areas for improvement and asked to repeat the assessment of competence on a later occasion. Irrespective of whether the minimum level is achieved, feedback on delivery will be given, with emphasis on where good performance has been achieved, as well as where improvement is possible, to both encourage and inform future delivery.

#### Information provision control intervention

The PN/HCA will give their patients an information pack of materials promoting walking produced by the British Heart Foundation and Walking the Way to Health. The PN/HCA will answer any questions the patient may have about walking more, and continue to provide usual care for that patient with regard to walking.

### Outcome measures

The main outcome measure will be mean daily number of minutes walked in the past week, assessed at all time points using the New Lifestyles NL-1000 Activity Monitor Pedometer (New-Lifestyles Inc, Lees Summit, Missouri, USA). This pedometer provides readings on number of minutes walked and step counts, over each of the previous seven days. The intensity threshold of each pedometer will be set to record movement on a setting of four or above (the range is 1 to 9), to assess physical activity of at least moderate intensity.

Psychological process will be assessed using one of two Theory of Planned Behaviour (TPB) [15] questionnaires, both of which measure several constructs. A longer 26-item version measures intentions, self efficacy/ PBC, attitudes, subjective norms, control beliefs and associated perceived power to inhibit/ facilitate behaviour in relation to walking for at least 30 minutes on average a day over the next 7 days [29]. A brief 6-item version of the questionnaire assesses only intentions, self efficacy/ PBC, attitudes, and subjective norms. Both versions of the TPB questionnaire are abbreviated versions of measures previously developed [10] and validated [13,14] in studies of walking with an adult volunteer general public sample.

Data on NHS resource use and personal costs to patients will be collected using standard measures for economic analysis [30]. Impact on Quality of Life will also be assessed using the EQ-5D questionnaire [31,32]. Comparing this with costs (directly and indirectly related to patient management) will enable incremental cost-effectiveness of the intervention to be estimated.

### Trial procedures

#### Practice recruitment

Practices in the relevant PCTs will be contacted by the study team, with the support of the local West Midlands (South) Primary Care Research Network (PCRN). Practices will be sent an invitation letter and short information sheet via the post, and this will be followed up by a telephone call. The research team will provide additional information to those practices that are interested, including a consent form for PN/HCAs, which provide a clear description of what is involved in participating in the trial. Practices will be randomised to the self-regulation intervention arm or to the information provision control arm. Randomisation will only proceed after the explicit agreement from the practice manager or a GP principal has been obtained, and the relevant PN/HCA has consented to delivering an intervention to 20 patients. Practices will be recruited on the understanding that if they are allocated to the control arm (which would still constitute good routine care), they will be offered training in the self-regulation intervention upon completion of study follow-ups.

#### Practice randomisation

Randomisation will be at the practice level, and will be stratified by practice size and index of deprivation scores, as assessed by practice postcode [33]. Strata will be defined according to whether the practices are above or below the median practice list size of 6127 for the four PCTs and whether the practice deprivation index is above or below the median deprivation index of 27.85, 14.57, 15.51 and 17.55 respectively for Coventry, Warwickshire, Worcestershire, and Herefordshire PCTs.

Randomisation will be performed by researchers at Coventry University external to the study team using materials prepared by the trial statistician (Stallard). These researchers have four lists, one for each of the four stratified groups: (a) small practices + high deprivation, (b) small practices + low deprivation, (c) large practices + high deprivation, (d) large practices + low deprivation. Each list contains a random ordering of "intervention" and "control" allocations. When the study team receive a consent form from a PN/ HCA to confirm that their practice will enter into the trial, the appropriate list will be consulted by the researchers external to the study team, who will record the practice name, and indicate whether they are intervention or control.

#### Patient recruitment

An initial list of patients will be selected from practice computerised records, using electronic searches, to identify eligible patients. A list of a random subsample of eligible patients (initially 200) will be sent to the GP or PN in each practice who will be nominated as responsible for referring patients. They will be given the inclusion criteria to guide recruitment.

Identified patients will be sent an invitation letter addressed from the practice, which emphasises that the intervention is intended for people who do less than the recommended 30 minutes of moderate or vigorous physical activity on at least five days a week. They will also be sent a patient information sheet explaining the study. Patients who are interested in taking part will be required to call the practice to make their first appointment with the PN/HCA delivering the intervention. Contact details for further information about the study will be included on the invitation letter and information sheet. Where the initial post to 200 patients does not yield 20 participating patients, further mail-outs to another random sample of eligible patients will be made.

#### Patient consent, receipt of intervention, and follow-up measurements

**Figure 1:**
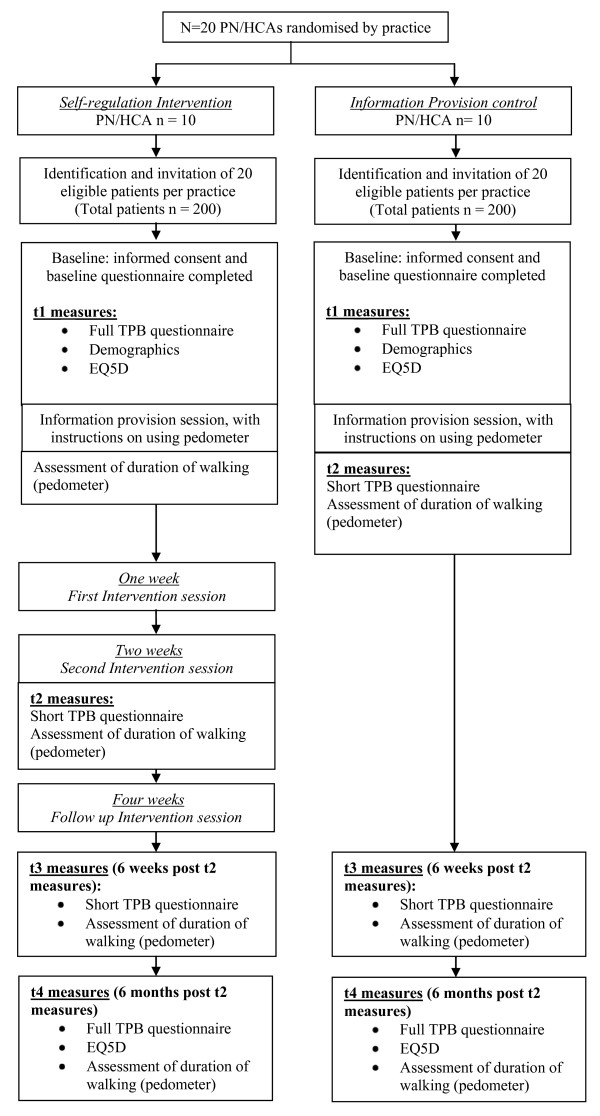
**Flowchart showing planned study recruitment numbers, and timing of intervention sessions and measurements**.

For both experimental conditions, each patient attends a face-to-face session at their own practice with the PN/HCA who is delivering the session. During this session, they are screened for whether they already do more than 30 minutes of moderate or vigorous physical activity per day on at least 5 days a week. If they are not already achieving this recommended level of physical activity, they are asked to provide informed consent, and complete the baseline (t1) questionnaire, which includes the full TPB measure, the EQ5D, and measures of ethnicity, employment status and education level. Information on patient date of birth, gender, and the clinical condition for which they were flagged (e.g. diabetes, hypertension, etc) will be recorded on a standardised form by PN/HCAs. The form will be sent to the research team, along with their contact details. Weight and height information for each patient will be recorded by the PN/HCA at baseline, which will allow calculation of body mass index. After this point, the procedure differs for patients in practices allocated to the self-regulation intervention condition, and patients in practices allocated to the information provision condition (see Figure 1).

For patients in practices allocated to the information provision control condition, the session will continue with delivery of the "information provision" intervention as described above: the PN/HCA will provide their patients with an information pack of materials promoting walking, and answer any questions the patient may have about walking more. At the end of this session, patients complete the post-intervention (t2) questionnaire, consisting of the brief TPB measure only, and are given a pedometer, with detailed instructions in its use. They are asked to wear it for a week, and return to the research team in a reply-paid envelope. This forms the first post-intervention assessment of walking.

For patients in practices allocated to the self-regulation intervention condition, the PN/HCA provides their patients with an information pack of materials promoting walking, and answers any questions the patient may have about walking more. Following this, two further face-to-face appointments will be booked, for delivery of the self-regulation intervention (as described above), at one week and two weeks after this initial session, plus the final intervention session four weeks after the initial session. At the end of this session, patients are given a pedometer, with detailed instructions for its use. They are asked to wear it for a week, and return it at their first intervention session where the PN/HCA will extract the data from the pedometer. This initial assessment of walking constitutes a pre-intervention measurement for those patients in the intervention arm.

Patients in practices allocated to the intervention condition then receive the self-regulation intervention, as detailed above. Sessions one and two will be tape-recorded, with the explicit agreement and signed consent of the patients involved. At the end of the second face-to-face session, patients complete the post-intervention (t2) questionnaire, and are given a pedometer, to wear for a week, and then return to the research team in a reply-paid envelope. This forms the first post-intervention assessment of walking.

Patients in practices allocated to the intervention condition receive a final follow-up intervention session, which may be by telephone or face-to-face, two weeks after their second face-to-face session. Despite this, the measurement procedures for patients in practices in both conditions are identical from t2 onwards.

At six weeks post intervention (i.e. six weeks after t2 for both groups), all patients are sent a questionnaire by post (t3) containing the brief TPB measure, and a pedometer, and asked to return these by post. At six months post intervention (t4), all patients are sent a questionnaire containing a full TPB measure, the EQ5D, and a final pedometer, which again they are asked to return by post.

To encourage retention, all participants who complete and return questionnaires or pedometers from t2 onwards will be sent postal feedback on their walking behaviour and psychological measurements in the form of a postal profile. Those who do not send back the pedometer at any measurement point will receive a reminder letter and follow-up telephone calls from the research team encouraging them to return these, as loss of numerous pedometers will be expensive, and threaten the validity of the study.

### Analysis

Planned intention-to-treat comparisons of the two groups at one week, six weeks and six months post-intervention (i.e. six months after t2 for both groups) will be conducted, with the primary dependent variable being mean daily walking, as assessed by pedometer. Mixed effects linear models will be fitted to compare the groups with a random practice effect to allow for clustering. The models will adjust for baseline characteristics at both practice and individual level. The former will include practice size and deprivation index, and the latter will include age, gender, BMI, ethnicity, employment status, educational level and clinical condition leading to referral.

As indicated above, the primary response will be the mean daily walking distance observed over up to seven days. Where less than five minutes walking is recorded for any particular day, the reading for that day will be treated as missing, and not used in calculating mean daily duration of walking. When individual days' data are missing, so long as data are available for two or more days, the mean will be taken over these non-missing days. If no data, or data from fewer than two days, are available for a particular patient at a particular timepoint, that assessment of walking will be treated as missing. These missing values will be imputed based on the baseline characteristics of the practice and individual [34]. A secondary analyses will be conducted excluding patients with missing data.

Planned exploratory sub-group analyses will be conducted to determine if the intervention has larger or smaller effects for groups defined in terms of age, ethnicity, gender, socio-economic status, or clinical condition, to provide information on any adverse impacts on social inequality.

#### Process evaluation

Formal mediation analyses will be conducted to identify the mechanisms by which the self-regulation intervention may work. If no clustering effects are found, the bootstrapping approach recommended by Preacher and Hayes will be used [35]. However, if clustering effects are present, then the Preacher and Hayes method would underestimate standard errors, so the Sobel [36] method will be used, with standard errors derived from the mixed effects model. In both instances, changes in TPB variables over the course of the intervention sessions (i.e. between t1 and t2) will be examined as potential mediators of effects on behaviour at t2. Changes in TPB variables over the course of the entire follow-up period (i.e. between t1 and t4) will be examined as potential mediators of effects on behaviour at t4.

#### Economic evaluation

Economic evaluation methods will, as far as possible, adhere to the recommendations of the NICE Reference Case [37]. The economic evaluation will consist of a within-trial analysis and economic modelling. Within trial analysis will compare direct costs and six month outcomes of patients randomised to intervention versus control. The perspective adopted will be that of the NHS and Public Social Services. A costing study will record intervention costs (to include staff time, capital, overheads and consumables), other NHS resource use (e.g. general practitioner visits) and patient expenditure. Unit costs for health care resources will be derived from local and national sources and performed in line with best practice [38]. Costs will be standardised to current prices where possible. Because of the short follow up period, we will not discount costs or benefits.

Comparison will be made between baseline and 6 months to estimate incremental cost-effectiveness ratios (ICERs) comparing the intervention with the control group in terms of the primary outcome measure (mean daily minutes walked in the past week) and costs [30]. Quality of life (EQ-5D) over the study period will be used to generate quality-adjusted life-years (QALYs) [39]. Outputs will be presented as ICERs, cost-effectiveness acceptability curves and expected net benefit. Sensitivity analyses will consider key cost drivers and factors that might affect the outcomes measured in order to explore uncertainty in the conclusions drawn.

## Discussion

The present study will provide a robust estimate of the effects of a brief walking intervention, when delivered by PN/HCAs in UK primary care, and the costs associated with any increases in walking produced. It will address the current lack of evidence for interventions that are effective at increasing walking, that can be offered to patients in primary care [7].

A key strength of this trial is that the evaluated intervention is built on extended developmental work. First, the intervention was developed based on explicit theory [15-17] evidence reviewing [14,23,24], and formative work [10-14]. Second, the intervention has demonstrated efficacy in producing large effects (d = 0.90) on objectively assessed walking of adult volunteers [14]. This effect has been replicated when delivered by a person who was not involved in the development of the intervention [21]. Third, the intervention has gone through a prolonged period of adaptation for delivery by PN/HCAs in primary care, to optimise fidelity of intervention delivery.

The main outcome of the developmental work in primary care was that the intervention was extended in duration from its initial single session format, to a multi-session format, which was more acceptable to both recipients and providers. There were slight changes to the intervention and training materials, to simplify these where they were unnecessarily complicated. The intervention materials and training used in the present trial were therefore acceptable to both recipients and providers. The fidelity of delivery to the intervention manual, as assessed at the completion of training has been consistently good: the PN/HCAs can deliver the intervention as intended when outside of routine primary care practice.

The main barrier to the successful delivery of the intervention by PN/HCAs in the developmental work undertaken in primary care has not been acceptability to recipients and providers: it has been the acceptability to primary care organisations. During the developmental work, particularly during the trial pilot, it has been difficult for practices to release the PN/HCAs for the required training, and to find time in consultation schedules for the delivery of the intervention. This is despite financial support from the PCRN and PCTs to fully reimburse practices for the time spent by PN/HCAs on training and delivery.

The developmental work highlights the importance of considering applicability and acceptability of behaviour change interventions to organisations, as well as to providers and recipients. This is consistent with the findings of a systematic review and meta-synthesis of qualitative studies that elicited views and experiences of nurses involved in the delivery of health behaviour change interventions in primary care [40]. The meta-synthesis inductively developed a conceptual framework of factors key to enhance delivery of behaviour change interventions by PNs. Although many of the factors identified had been well described by previous writing on fidelity generally [25], the influence of healthcare organisations on fidelity of delivery was clearly essential, but had previously received little consideration.

## Conclusion

The developmental work has adapted an intervention which has demonstrated efficacy with adult volunteers, for delivery by PN/HCAs in primary care. This follows the MRC framework for development and evaluation of complex interventions [8,9], to optimise the chance of the intervention demonstrating efficacy when delivered to primary care patients. If the trial finds the intervention is effective at increasing walking, then the training programme will be disseminated more widely throughout primary care, to the benefit of the target population. However, if the trial finds no effect, the statistical mediation analyses and explorations of fidelity in the exploratory (pilot) trial described should identify why it did not demonstrate efficacy. Potential reasons include that the intervention was not delivered as intended, or that it was delivered as intended, but failed to alter the hypothesised mediators, or that it did alter the hypothesised mediators but these failed to translate into behaviour change.

## Abbreviations

EQ5D: EuroQol Group health status measure; HCA: Health Care Assistant; ICER: Incremental Cost-Effectiveness Ratio; MRC: Medical Research Council; PBC: Perceived Behavioural Control; PCRN: Primary Care Research Network; PCT: Primary Care Trust; PN: Practice Nurse; QALY: Quality Adjusted Life Year; TPB: Theory of Planned Behaviour.

## Competing interests

The authors declare that they have no competing interests.

## Authors' contributions

DPF is the Principal Investigator who initially conceived and designed the study with JD and SM. DPF additionally oversaw intervention and training development, and led the funding application and the writing of this manuscript. SLW coordinated the study, including producing study procedures, and developed and refined intervention and training materials. CT contributed to study procedures, especially those relating to intervention fidelity. SLW, DPF and CT ran the provider training. CT, SLW, DPF and JD designed interview schedules with PN/HCAs and patients; interviews were conducted by CT and SLW. SM additionally contributed to the design of training procedures and fidelity issues. JD led on primary care issues, and contributed to recruitment. NS contributed to study design and led on statistical analysis. AS led on health economics, and contributed to measure selection. All authors read and approved the final manuscript.

## Pre-publication history

The pre-publication history for this paper can be accessed here:

http://www.biomedcentral.com/1471-2296/12/56/prepub
